# Shear Strength of Brackets Bonded with Universal Adhesive Containing 10-MDP after 20,000 Thermal Cycles

**DOI:** 10.1155/2020/4265601

**Published:** 2020-02-17

**Authors:** Mariana Almeida Mello Proença, Karime Tavares Lima da Silva, Alisson Costa e Silva, Edilausson Moreno Carvalho, José Bauer, Ceci Nunes Carvalho

**Affiliations:** ^1^University Pitágoras, School of Dentistry, São Luís, 65065-470, Maranhão, Brazil; ^2^Department of Restorative Dentistry, School of Dentistry, Ceuma University (Uniceuma), São Luis, Maranhão, Brazil; ^3^Federal University of Maranhão (UFMA), Discipline of Dental Materials, School of Dentistry, Av. dos Portugueses, 1966, Zip Code 65080-805, São Luis, Maranhão, Brazil

## Abstract

**Objectives:**

The aim of this study was to evaluate the shear bond strength of metal brackets bonded with different universal adhesive systems containing 10-MDP and Transbond Plus Self Etching Primer after 20,000 thermal cycles. *Materials and Methods*. A total of 130 sound bovine teeth were used, which are divided into 5 groups (*n* = 26) according to the adhesive system used: All-Bond Universal (Bisco), Ambar Universal (FGM), Clearfil Universal Bond (Kuraray), Single Bond Universal (3M/ESPE), and Transbond Plus SEP (3M/ESPE) as control. The adhesives were applied for 20 seconds and bonded with a resin Transbond XT (3M/ESPE). After this, the teeth were submitted to 20,000 cycles at 5°C and 55°C. Afterwards, the shear bond strength test was performed in a universal test machine (Instron 3342). The adhesive remnant index (ARI) was evaluated under a stereomicroscope at 10x magnification and scanning electronic microscopy (SEM, Hitachi 3030). The shear bond strength data were submitted to One-Way ANOVA (*α* = 0.05) and the ARI to the Kruskal–Wallis test (*α* = 0.05) and the ARI to the Kruskal–Wallis test (

**Results:**

Statistical analysis showed that the universal adhesive systems presented mean shear bond strength values similar to Transbond Plus SEP (*p* < 0.05). The universal adhesive presented similar ARI values among them but differed from those of Transbond Plus SEP (*p* < 0.05). The universal adhesive presented similar ARI values among them but differed from those of Transbond Plus SEP (

**Conclusions:**

The results show that universal adhesive systems may be used for bonding metal brackets if the orthodontist wants to maintain dental enamel health.

## 1. Introduction

Acid etching of enamel has been a technique used successfully since its introduction in 1955, by Buonocore who recommended the use of phosphoric acid at a concentration of 85% for 30 seconds [1]. This procedure creates micropores on the enamel surface, into which resin tags are incorporated, forming a micromechanical bond between the enamel and resin [1]. The formation of long resin tags penetrating into enamel and forming a resistant monoblock is desirable in restorative treatments.

On the contrary, orthodontic treatment with fixed appliances is a temporary procedure. In this sense, when the enamel is etched with phosphoric acid, the formation of long resin tags may lead to some harm to the dental substrate: crack and microfracture formation [2], staining [3], reduction in the modulus of elasticity, and enamel hardness [4].

In the year 2008, an estimated 29% of dentists in the United States used self-etching orthodontic adhesive systems [5]. This could be justified by factors such as reduction in chair time and technique sensitivity since the application of this material was fast and performed in a lower number of steps [5]. Furthermore, these materials promised to cause fewer irreversible changes in enamel than the conventional systems because they had a reduced capacity for penetrating into the substrate [6]. An important representative of this category is the methacrylated ester-phosphoric acid-based adhesive system. However, one of the reasons for the excellent clinical performance of this material [7] is its elevated acidity (pH ≤ 1), that provides micromechanical retention of the resin tags in enamel [8] as phosphoric acid.

Recently, “universal” or “multimode” adhesive systems were launched on the market; these materials had a less aggressive pH (2 < pH < 3) and performed bonding by means of functional monomers [8]. These materials could be applied to the substrate either previously etched or without etching [5, 9–11]. This is due to the presence of acidic functional monomers that have a high affinity for the calcium of hydroxyapatite [12].

Previous studies have confirmed that 10-MDP (methacryloyloxydecil dihydrogen phosphate) is at present the great acidic functional monomer because it establishes a stable and lasting interaction with both dentin and enamel [8, 11]. The presence of 10-MDP in the adhesive system enables bracket bonding without the need for micromechanical retentions that are harmful to the integrity of enamel.

Several thermal cycles can cause a stress in the adhesive interface (metal bracket, adhesive, and tooth). The difference of the coefficients of thermal expansion between the substrates involved can cause premature debonding of the brackets [13].

Therefore, the aim of the present study was to evaluate the shear bond strength of metal brackets bonded with universal adhesive systems (All-Bond Universal, Ambar Universal, Clearfil Universal Bond, and Single Bond Universal) compared with Transbond Plus SEP. The authors established the null hypothesis that there would be no significant difference in the bond strength promoted by Transbond Plus SEP and by the universal adhesive systems.

## 2. Materials and Methods

### 2.1. Sample Calculation

To calculate the sample size, the software G*∗*Power 3.0.10 (Franz Faul, Universität Kiel, Germany) was used. Based on a similar study [14], an *α* error = 0.05 and test power of 80% were considered so that 26 specimens per group would be required to detect possible differences, totaling 130 specimens. Bovine incisors [15] free of enamel defects were stored for 1 month in a 0.1% thymol solution to control bacterial growth [16]. Metal brackets for maxillary incisors (Roth 022, Kirium, 3M Unitek, Monrovia, California, USA) were used in this study.

### 2.2. Preparation of Specimens

Before bonding, the vestibular surfaces of the teeth were cleaned with pumice stone and water for 15 seconds, by using a rubber cup. The teeth were washed and dried. The materials used in this study and their compositions are presented in Table 1. The adhesive systems were applied for 20 seconds in the central region of the vestibular surface of the teeth; a light, smooth jet of air was applied for 1-2 seconds; and they were polymerized for 10 seconds (Radii-cal, 1200 mW/cm^2^, SDi, Victoria, Australia). The resin composite (Transbond XT, 3M Unitek) was applied at the base of the bracket (Roth 022, Kirium, 3M Unitek, Monrovia, California, USA), and it was seated on the vestibular surface of the tooth with a tensiometer (Odeme Technology, São Paulo, Brazil) with a load of 300 mg for 10 seconds to guarantee a uniform layer of resin [17]. Light activation was performed on two surfaces (mesial and distal) for 20 seconds each (Radii-cal, 1200 mW/cm^2^, SDi, Victoria, Australia).

The specimens were submitted to 20,000 thermal cycles (Thermocycle, Biopdi, São Carlos, Brazil) simulating two years of treatment. Each cycle had a duration of 60 seconds: 30 seconds submerged in a tub with water at 5°C and 30 seconds submerged in a tub with water at 55°C [18].

### 2.3. Shear Bond Strength

**Figure 1:**
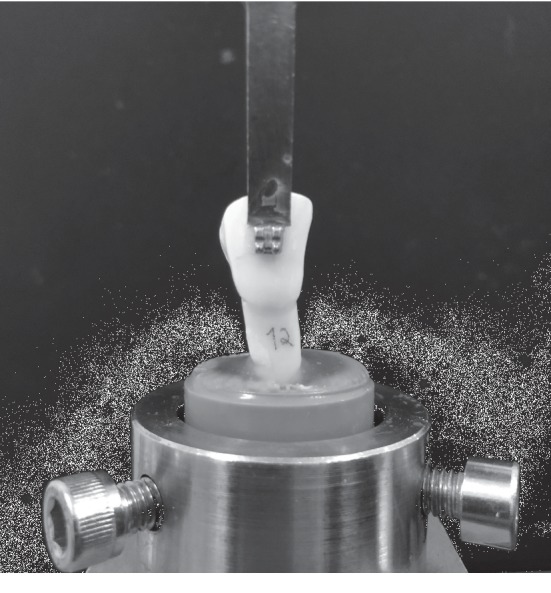
Specimen block mounted in the universal test machine.

The teeth were embedded in PVC tubes in acrylic resin, with only the coronal part remaining visible. In addition, they were positioned in such a way that the brackets remained parallel to the vertical plane (Figure 1). The set was taken to the universal test machine (Instron 3342, Canton, USA), and an occlusal-gingival load was applied at a speed of 1.0 mm/min [19]. The force required to debond the bracket was recorded in Newton (N) and converted to mega-Pascal in the ratio of Newtons to surface area of the bracket (MPa = N/mm^2^).

### 2.4. Adhesive Remnant Index (ARI)

After the brackets were debonded, the teeth were visually evaluated under a stereomicroscope (Kozo Optical and Electronic Instrumental, China) at 10x magnification for classification of the adhesive remnant index (ARI) in the following manner: 0 = absence of composite on the tooth; 1 = less than a half of composite on the tooth; 2 = more than a half of composite on the tooth; and 3 = all of the composite on the tooth with an impression of the bracket base [20]. A scanning electron microscope (Hitachi, TM3030, Tokyo, Japan) was used to evaluate the enamel condition in 2 specimens with each adhesive according to the different fracture patterns found after bracket debonding.

### 2.5. Statistical Analysis

The SigmaPlot 13.0 software (Systat Software Inc., San Jose, USA) was used. To evaluate the normality of the data, the Shapiro–Wilk test was performed. The shear bond strength data were submitted to One-Way ANOVA and Holm-Sidak post hoc test (*α* = 0.05). The ARI data were submitted to the Kruskal–Wallis and post hoc Dunn's test (*α* = 0.05).

## 3. Results

**Figure 2:**
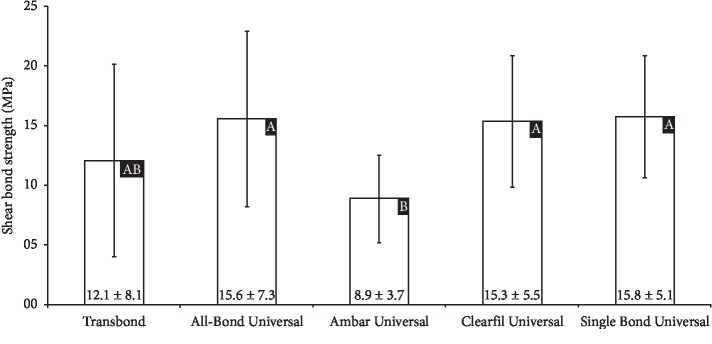
Shear bond strength test results (mean and standard deviation) of the adhesive systems. ^∗^Different letters correspond to statistical difference (*p* < 0.05).

The mean shear bond strength (MPa) of each group is demonstrated in Figure 2. Statistical analysis demonstrated that there was no difference in the shear bond strength values of the universal adhesives when compared with those of Transbond Plus SEP (*p* < 0.01).

**Figure 3:**
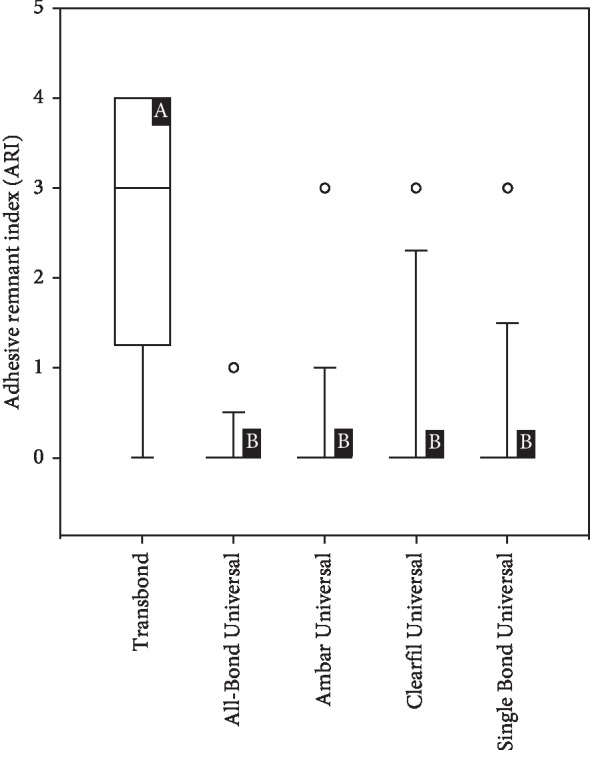
Adhesive Remnant Index (ARI) values of the adhesive systems.^∗^Different letters correspond to statistical difference (*p* < 0.05).

**Figure 4:**
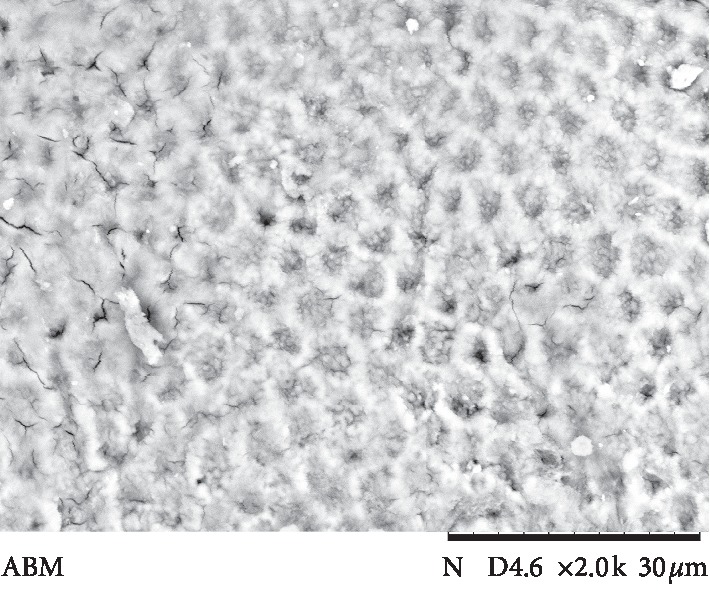
Scanning electron microscopy view of enamel surface etched with a universal adhesive system (All-Bond Universal system) (magnification: 2000x; bars 30 *μ*m) showing a pattern of regularity and a light removal of the prism nucleus (arrows).

**Figure 5:**
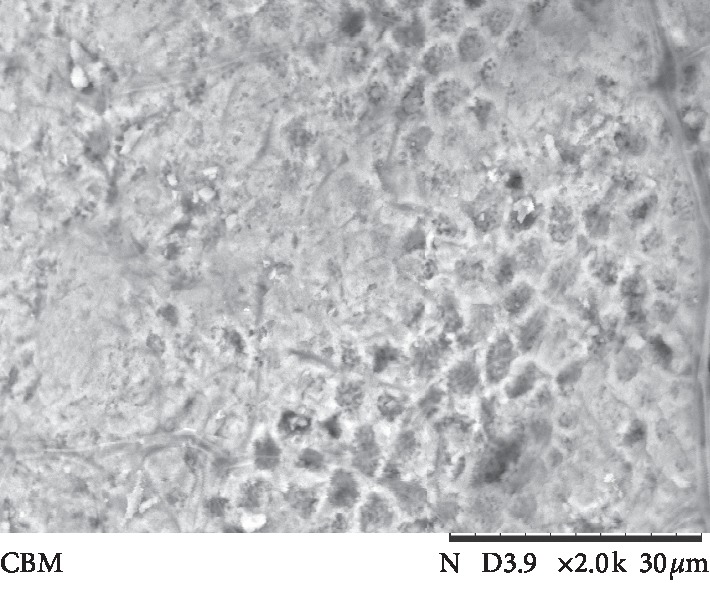
Scanning electron microscopy view of enamel surface etched with a universal adhesive system (Ambar Universal system) (magnification: 2000x; bars 30 *μ*m) showing intact areas (arrows) and light conditioning areas (asterisks).

**Figure 6:**
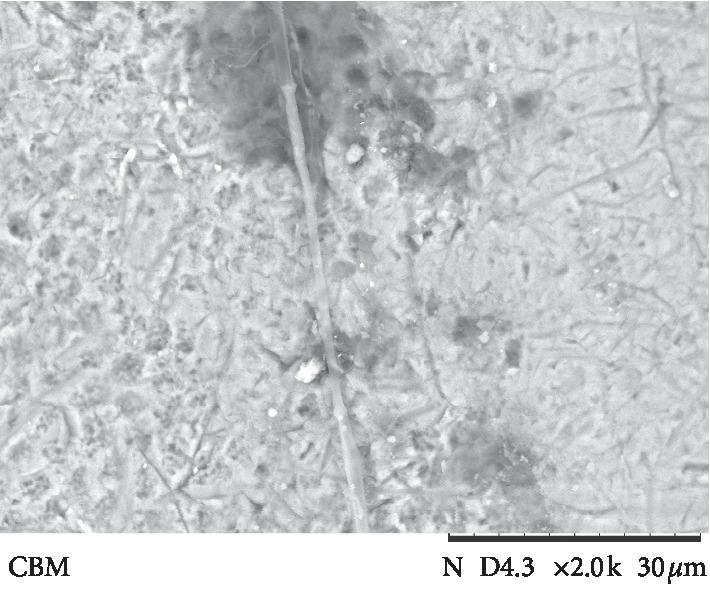
Scanning electron microscopy view of the enamel surface etched with a universal adhesive system (Clearfil Universal Bond) (magnification: 2000x; bars 30 *μ*m). Its possible observes absence of a pattern in conditioning, with intact areas (arrows), areas of light conditioning (asterisks), and deep conditioning (circle).

**Figure 7:**
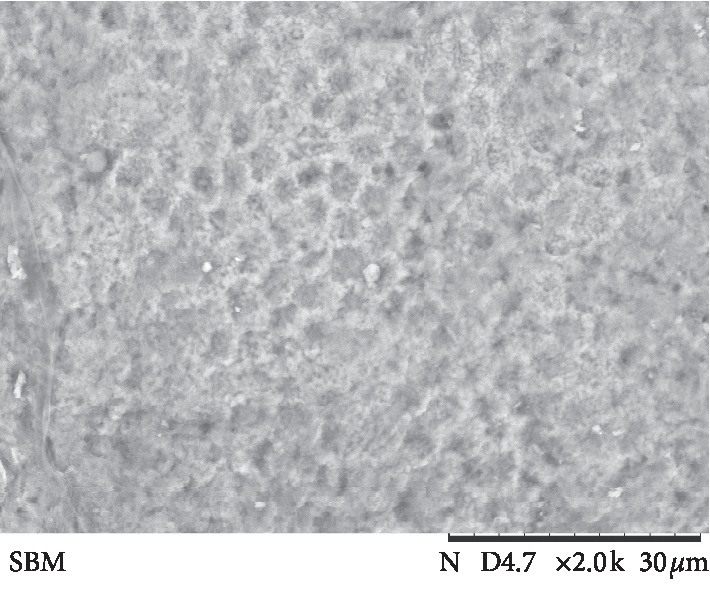
Scanning electron microscopic view of the enamel surface etched with a universal adhesive system (Single Bond Universal) (magnification: 2000x; bars 30 *μ*m) showing a light removal of the prism nucleus (arrows).

**Figure 8:**
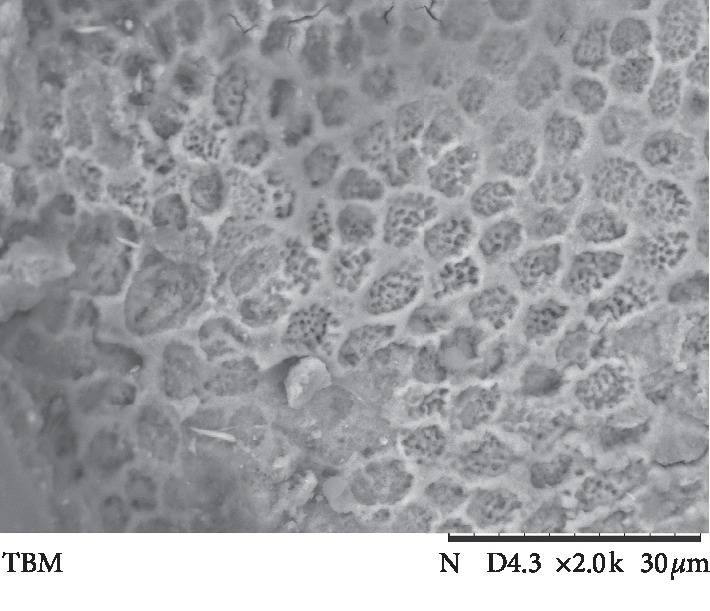
Scanning electron microscopic view of the enamel surface etched with a self-etching adhesive system (Transbond Plus Self-Etching Primer) (magnification: 2000x; bars 30 *μ*m). A regular conditioning pattern, with formation of micropores in the depth of the prism nucleus (arrows), is observed.

The results of ARI values are presented in Figure 3. Failure mode analysis pointed out a difference in ARI between the universal adhesive systems and Transbond Plus SEP (*p* < 0.001), which presented the highest adhesive remnant index values. Figures 4–8 show pattern of enamel demineralization caused by adhesive systems used in the present study.

## 4. Discussion

The present study evaluated the bond strength of universal adhesive systems used for bracket bonding. Some self-etching adhesive systems (Transbond Plus SEP) were developed for use in orthodontics, especially for bracket bonding. This material performs enamel etching by means of acidic monomers that guarantee a low pH [10], whereas the universal adhesive systems containing MDP were developed to meet the demands of innumerable specialties in dentistry to be applied in diverse manners and to different substrates (enamel and dentin). Although they acted by means of different mechanisms, we found no differences between the shear bond strength of the orthodontic self-etching (Transbond Plus SEP) and the universal adhesive systems containing 10-MDP; thus, we accepted the null hypothesis.

The mean values of shear bond strength reported in the present study ranged between 8.9 and 15.8 MPa. In the literature, there are not clear guidelines about shear force limits. Scribante et al. highlighted that a good orthodontic biomaterial should allow good adhesion in order to sustain masticatory forces (5–10 MPa), and adhesion forces should not be too strong in order to avoid enamel loss after debonding (40–50 MPa). Thus, despite these limits are mostly theoretical, the ideal orthodontic biomaterial should have bonding forces included in the interval of 5–50 MPa.

The good performance of the universal adhesive systems has been attributed to the presence of 10-MDP in their composition. This monomer forms nanolayers at the bond interface, where the calcium ions released after partial dissolution of the hydroxyapatite binding together to form the Ca-MDP bond that is highly stable [21]. This chemical affinity is responsible for low rates of dissolution of calcium salts, resulting in an optimum performance of these materials in the bond strength tests [13]. On the contrary, the bond strength of the self-etching adhesive (Transbond Plus SEP) is attributed to the presence of a strong acid in its composition, responsible for promoting etching over 5 *µ*m deep on the enamel surface, a behavior very similar to that of phosphoric acid [8].

However, the less-aggressive adhesive systems may be successfully used for bracket bonding since a low pH was not related to high bond strength values [22]. In addition to the higher level of enamel demineralization, the low pH of the self-etching adhesive system (Transbond Plus SEP) enables greater penetration of the resin tags into enamel [22]. SEM analysis confirms a large demineralization potential of the self-etching adhesive system, forming micropores in the depth of prism nucleus (arrows in Figure 8), and the low demineralization pattern of 10-MDP-containing universal adhesive systems, with intact or light demineralized areas in the substrate (arrows in Figures 4–7).

Clinically, complete removal of the remnants of resin material from the enamel surface may lead to loss of health tooth structure. Moreover, the permanence of these resin remnants may compromise esthetics [23], causing microfractures [24] and increasing the surface roughness [25], which favors bacterial plaque retention [26]. Therefore, it is important to have a material that promotes satisfactory bond strength to maintain the bracket on the enamel surface during orthodontic treatment without causing irreversible damage to the enamel structure [10].

When considering the ARI, even if methods of measurement could influence score assignment results [27], ARI score is nowadays widely used in bonding studies to assess and discuss adhesive left on tooth surface after debonding. Generally, a score of “0” is often related to contaminants over enamel. The ARI score of “3” means that polishing procedures are longer as more adhesive remains on tooth surface. Therefore, an orthodontic biomaterial should aspire to a mixed adhesion modality (ARI “1” and “2”).

The universal adhesive systems containing 10-MDP demonstrated efficient bond of the bracket to the tooth, without the presence of adhesive remainders on the substrate, according to the adhesive remnant index analysis made in this study (Figure 2). Retention of the resin tags was more prevalent in the self-etch group that presented the majority of specimens with ARI 3, while the universal adhesives predominantly presented ARI 0. This involves dispensing with the use of rotary instruments, reducing the risks of damage to enamel and shorter chair time [28]. However, a direct application in clinical dentistry of the results of the present study is not applicable, as it is an in vitro study. Further clinical randomized controlled trials should be carried out in order to confirm the outcomes of the present report.

Exposure of the materials to 20,000 thermal cycles may also have been important for the similarity in the bond strength results presented. Previous studies have demonstrated a significant reduction in bond strength after 10,000 and 20,000 thermal cycles [18]. Fujita et al. [10] observed that there was no difference in the bond strength promoted by an adhesive system containing 10-MDP after 20,000 thermal cycles. This is because the monomer MDP plays an important role in the durability of the bond to enamel, by inhibiting hydrolysis and impeding stress due to fatigue [29].

## 5. Conclusions

Thus, it is possible to conclude that the indication of universal adhesives containing 10-MDP for bonding orthodontic brackets could be feasible in terms of shear bond strength because they provided good bond strength without damage to the enamel.

## Figures and Tables

**Table 1:** Materials used in this study and their compositions.

Adhesive system	pH	Composition^∗^
All-Bond Universal (Bisco Inc., USA)	2, 5–3, 5	Bis-GMA, 10-MDP, HEMA, ethyl-4-dimethylaminobenzoate, ethanol, and water
Âmbar Universal (FGM, Brazil)	2, 6–3	10-MDP, UDMA, HEMA, methacrylated hydrophilic monomers, camphorquinone, silanized silica, ethyl 4-dimethylaminobenzoate, and ethanol
Clearfil Universal Bond (Kuraray Noritake, Japan)	2, 3	Bis-GMA, 10-MDP, HEMA, camphorquinone, ethanol, colloidal silica, aliphatic hydrophilic dimethacrylate, and water
Single Bond Universal (3M ESPE, USA)	2, 7	Bis-GMA, 10-MDP, HEMA, dimethacrylate, vitrebond copolymer, filler, initiators, silane, ethanol, and water
Transbond Plus SEP (3M Unitek, USA)	0-0, 5	Mono-HEMA phosphate, Di-HEMA phosphates, water, camphorquinone, methacrylated pyrophosphate, ethylene dimethacrylate, orthophosphoric acid, tris (2-(methacryloyloxy) ethyl) phosphate

^∗^Compositions according to information obtained from their respective manufacturers. Bis-GMA: bis-phenol A glycidyldimethacrylate, UDMA: urethane dimethacrylate, HEMA: 2-hydroxyethyl methacrylate, and 10-MDP: 10-methacryloyloxydecyl dihydrogen phosphate.

## Data Availability

The Excel file (bonding strength and statistical analysis (PDF)) data used to support the findings of this study are included within the supplementary information files.

## Supplementary Materials

Supplementary Materials Excel (Adhesive Universal Bracket) file: the shear bond strength data of all tested groups (spreadsheet: bonding Bracket) and also the fracture mode of the evaluated groups (spreadsheet: ARI). PDF file (ARI): the statistical analysis of fracture mode data from the tested study groups. PDF file (Bracket One-Way Analysis of Variance): the statistical analysis of the shear bond strength data of the tested study groups.
