# Supplementary material for: Shear Strength of Brackets Bonded with Universal Adhesive Containing 10-MDP after 20,000 Thermal Cycles
Source: Int J Dent. 2020 Feb 17;2020:4265601. doi: 10.1155/2020/4265601 (PMC7048917; doi:10.1155/2020/4265601)
Supplement: Supplementary Materials — Excel (Adhesive Universal Bracket) file: the shear bond strength data of all tested groups (spreadsheet: bonding Bracket) and also the fracture mode of the evaluated groups (spreadsheet: ARI). PDF file (ARI): the statistical analysis of fracture mode data from the tested study groups. PDF file (Bracket One-Way Analysis of Variance): the statistical analysis of the shear bond strength data of the tested study groups. [file 4265601.f1.zip › 4265601.f1/ARI.pdf]

One Way Analysis of Variance sexta-feira, novembro 18, 2016, 09:57:19

Data source: Data 1 in Notebook1

Normality Test (Shapiro-Wilk): Failed (P < 0,050)

Test execution ended by user request, ANOVA on Ranks begun

Kruskal-Wallis One Way Analysis of Variance on Ranks sexta-feira, novembro 18, 2016, 09:57:19

Data source: Data 1 in Notebook1

**Group**	**N**	**Missing**	**Median**	**25%**	**75%**
Transbond	24	0	3,000	1,250	4,000
Allbond	24	0	0,000	0,000	0,000
Ambar	26	0	0,000	0,000	0,000
Clearfil	26	0	0,000	0,000	0,000
Single Bond	24	0	0,000	0,000	0,000

H = 52,267 with 4 degrees of freedom. (P = <0,001)

The differences in the median values among the treatment groups are greater than would be expected by chance; there is a statistically significant difference (P = <0,001)

To isolate the group or groups that differ from the others use a multiple comparison procedure.

All Pairwise Multiple Comparison Procedures (Dunn's Method) :

**Comparison**	**Diff of Ranks**	**Q**	**P**	**P<0,050**
Transbond vs Allbond	48,938	4,717	<0,001	Yes
Transbond vs Single Bond	47,854	4,612	<0,001	Yes
Transbond vs Ambar	42,989	4,226	<0,001	Yes
Transbond vs Clearfil	42,239	4,152	<0,001	Yes
Clearfil vs Allbond	6,699	0,658	1,000	No
Clearfil vs Single Bond	5,615	0,552	1,000	Do Not Test
Clearfil vs Ambar	0,750	0,0752	1,000	Do Not Test
Ambar vs Allbond	5,949	0,585	1,000	Do Not Test
Ambar vs Single Bond	4,865	0,478	1,000	Do Not Test
Single Bond vs Allbond	1,083	0,104	1,000	Do Not Test

Note: The multiple comparisons on ranks do not include an adjustment for ties.
