# Supplementary material for: Shear Strength of Brackets Bonded with Universal Adhesive Containing 10-MDP after 20,000 Thermal Cycles
Source: Int J Dent. 2020 Feb 17;2020:4265601. doi: 10.1155/2020/4265601 (PMC7048917; doi:10.1155/2020/4265601)
Supplement: Supplementary Materials — Excel (Adhesive Universal Bracket) file: the shear bond strength data of all tested groups (spreadsheet: bonding Bracket) and also the fracture mode of the evaluated groups (spreadsheet: ARI). PDF file (ARI): the statistical analysis of fracture mode data from the tested study groups. PDF file (Bracket One-Way Analysis of Variance): the statistical analysis of the shear bond strength data of the tested study groups. [file 4265601.f1.zip › 4265601.f1/Bracket (One Way Analysis of Variance).pdf]

One Way Analysis of Variance sexta-feira, novembro 04, 2016, 10:09:32

Data source: Data 1 in Mariana

Normality Test (Shapiro-Wilk): Passed (P = 0,086)

Group Name	N	Missing	Mean	Std Dev	SEM
Transbond	24	0	12,063	8,060	1,645
Single Bond	23	0	15,757	5,128	1,069
Clearfil	26	0	15,341	5,671	1,112
All Bond	26	0	15,574	7,438	1,459
Ambar	26	0	8,862	3,726	0,731

Source of Variation	DF	SS	MS	F	P
Between Groups	4	926,019	231,505	6,030	<0,001
Residual	120	4606,901	38,391		
Total	124	5532,919			

The differences in the mean values among the treatment groups are greater than would be expected by chance; there is a statistically significant difference (P = <0,001).

Power of performed test with alpha = 0,050: 0,958

All Pairwise Multiple Comparison Procedures (Holm-Sidak method):
Overall significance level = 0,05

Comparisons for factor:

Comparison	Diff of Means	t	P	P<0,050
All Bond vs. Ambar	6,712	3,906	0,002	Yes
Single Bond vs. Ambar	6,895	3,888	0,001	Yes
Clearfil vs. Ambar	6,479	3,770	0,002	Yes
Single Bond vs. Transbond	3,694	2,043	0,266	No
All Bond vs. Transbond	3,511	2,002	0,253	No
Clearfil vs. Transbond	3,278	1,869	0,282	No
**Transbond vs. Ambar**	**3,201**	**1,825**	**0,254**	**No**
Single Bond vs. Clearfil	0,416	0,234	0,994	No
All Bond vs. Clearfil	0,233	0,136	0,988	No
Single Bond vs. All Bond	0,183	0,103	0,918	No
